# Tracing of Helicobacter Pylori in Patients of Otitis Media with Effusion by Polymerase Chain Reaction

**Published:** 2015-05

**Authors:** Mahmood Shishegar, Mohammad Motamedi-Far, Seyed Basir Hashemi, Abbas Bigham-Sadegh, Amir Emami

**Affiliations:** 1 Department of Otolaryngology, School of Medicine, Shiraz University of Medical Science, Shiraz, Iran;; 2 Department of Microbiology, School of Medicine, Shiraz University of Medical Science, Shiraz, Iran

**Keywords:** Serous otiti, Helicobacter pylori, Polymerase chain reaction

## Abstract

Otitis media with effusion (OME) is one of the most common causes of hearing loss (HL) in children. It has been reported that several factors such as eustachian tube dysfunction, insufficiencies in the aeration of the mastoid cells, allergies, immunity, and infections play an important role in the etiology of the disease. Little is known about the role of Helicobacter pylori (H. pylori) in extragastric diseases. Because of the near location of the nose, sinuses, tonsils, and adenoids to the eustachian tube and middle ear, we believe it is possible to have H. pylori in the middle ear. The present study was designed to investigate the presence of H. pylori by polymerase chain reaction (PCR) in the middle ear effusion of patients with OME.

The study was performed on 21 patients, 19 patients were affected bilaterally, and 2 patients were affected unilaterally, from which 40 specimens were collected. OME was diagnosed through findings by otoscopic examination and tympanogram. The middle ear fluid samples were collected under sterile conditions. A total of 40 samples was stored at -80°C until analyzed by PCR assay. From 40 specimens, 2 specimens were serosal and 38 specimens were mucoid.

PCR results of the study in assays for Helicobacter pylori were not positive in all collected specimens. Overall, probably there was no H. pylori organism in free-floating form and thus could not be detected by PCR.

## Introduction

Otitis media with effusion (OME) is one of the most common causes of hearing loss (HL) in children. It has been reported that several factors such as eustachian tube dysfunction, insufficiencies in the aeration of the mastoid cells, allergies, immunity, and infections play an important role in the etiology of the disease.^1^ Recent studies have investigated the possibility of a causal relationship between OME and the presence of Helicobacter pylori in the middle ear.^2^

There has been a heightened interest in recent literature on the role of H. pylori as one of the organisms implicated in otitis media with effusion (OME). Because H. pylori has been discovered in anatomical sites such as the adenoids, which are in proximity to the Eustachian tube, it naturally follows that this bacterium may play a role in middle ear disease.^2^ It has been reported that gastroesophageal reflux can be related to some problems such as laryngitis, pharyngitis, rhinosinusitis, eustachian tube dysfunction, recurrent otitis media, and otitis media with effusion. In OME patients, pepsinogen and pepsin were detected in the middle ear fluid and the possibility of gastric fluid presence in the middle ear and its role in pathogenesis of OME was proposed.^3^ The aim of this study is to trace Helicobacter pylori in patients of otitis media with effusion by polymerase chain reaction.

## Patients and Methods

*Patients and Sample Collection*

The study was performed on 21 patients with OME, admitted to the ENT department of Khalili Hospital (Shiraz, Iran) from April 2012 to June 2012. The study group consisted of 9 girls and 12 boys with a mean age of 5.57±2.24 (Mean±SD) and a range of 2 to 12 years. 19 patients were affected bilaterally and 2 patients were affected unilaterally, from which 40 specimens were collected. OME was diagnosed through findings by otoscopic examination and tympanogram. The inclusion criteria were the presence of middle ear effusion for more than 3 months, not being on antibiotic treatment for at least 2 weeks, and not having other medical problems or history of otologic surgery. The exclusion criteria were, having had antibiotic treatment for at least 2 weeks or other medical or anatomical problems such as cleft palate or positive history of otologic surgery. Informed consent was obtained from parents before inclusion in the study in accordance with the ethical standards of the local Ethics Committee of Shiraz Medical University. The patients included in the study were examined for hearing loss, fullness in the ear, tinnitus, and ear pain. In addition, whether paracentesis or ventilation tube was performed recurrently was asked. In all cases, a myringotomy operation (with placement of a ventilation tube) was carried out. Effusions were obtained by myringotomy under general anesthesia and with the help of a Zeiss Opmi-6 microscope (Zeiss Company, Germany). For this purpose, the outer ear canal was cleaned with 70% alcohol solution. After cleansing the outer ear canal, myringotomy was performed in the lower quadrant of the tympanic membrane with a paracentesis knife. In bilateral cases, middle ear fluid samples from both ears were taken. Middle ear fluid was transferred to a collector tube attaching to suction tube. The middle ear fluid samples were collected under sterile conditions. A total of 40 samples was stored at -80°C until analyzed by PCR assay.

*Detection of Urease Gene for Detection of H. pylori with PCR*

Samples were extracted with AccuPrep® Genomic DNA Extraction Kit (Bioneer, Korea) according to the manufacturer’s instructions. To evaluate the presence of H. pylori in the extracted samples, the PCR was performed with the specific primers targeted urease gene regions according to table 1.^4^

**Table 1:** Primer sequence target genes, product size, and PCR protocol

***H. pylori urease C*** ** gene primer **	** Sequence (5^’^-3^’^ direction) **	**Cycle profile (35×)**	**Product size**
Urease C-F	TGGGACTGATGGCGTGAGGG	95ºC/1min-57ºC/1min-72ºC/1min	820 bp
Urease C-R	AAGGGCGTTTTTAGATTTTT	95ºC/1min-57ºC/1min-72ºC/1min	820 bp

The PCR was performed in 50 µl reaction volume in a Eppendorf Thermocycler (Germany). Reaction mixtures contained 0.4 µM (each) primer, 0.2 µM (each) deoxynucleoside triphosphate (dATP, dGTP, dTTP and dCTP ) from CinnaGen-Iran, and 1X PCR reaction buffer 1.5 µM, Mgcl2 with the addition of 10 µl of the extracted DNA.

DNA from *H. pylori* ATCC 53726 (confirmed clinical strain) and a tube containing water in place of DNA was assayed in each PCR run as positive and negative controls respectively of the reaction.

Ten-microliter of each PCR products were subjected to gel electrophoresis (1.5% agarose) in Tris-acetate-EDTA (TAE) buffer for one hour. The gel was stained in ethidium bromide solution (1 µg/ml) for 10 minutes. The results were analyzed according to the product size, which were visualized in gel documentation system (UVItec, UK) and photographed. 

## Results

**Figure 1:**
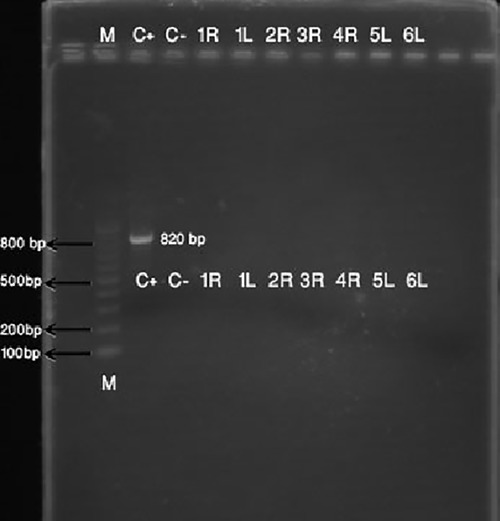
Results of PCR for detection of H. pylori in middle ear effusion of patients with OME. C+: Positive control; C-: Negative control; 1R, 1L, 2R, 3R, 4R, 5L and 6L are patient’s specimens that were all negative

From 40 specimens, 2 specimens were serosal and 38 specimens were mucoid. PCR results of the study are given in table 2. PCR assays for *Helicobacter pylori *were not positive in all collected specimens in our study (figure 1).

**Table 2:** Characteristics of patients with OME evaluated for H. pylori in middle ear effusion

**Patient No**	**Age (Year)**	**Sex**	**Middle ear effusion**	**Nature of effusion fluid**	**Tympanometry**	**H. Pylori**
1	4	F	Bilateral	Mucoid	B type	-
2	5	M	Unilateral	Mucoid	B type	-
3	6	F	Bilateral	Serous	B type	-
4	6	M	Bilateral	Mucoid	B type	-
5	12	M	Bilateral	Mucoid	B type	-
6	8	F	Bilateral	Mucoid	B type	-
7	3	M	Bilateral	Mucoid	B type	-
8	5	F	Bilateral	Mucoid	R=B type L=C type	-
9	4	F	Bilateral	Mucoid	B type	-
10	4	F	Bilateral	Mucoid	B type	-
11	6	M	Bilateral	Serous	R=B type L=C type	-
12	5	M	Bilateral	Mucoid	B type	-
13	4	F	Bilateral	Mucoid	C type	-
14	6	F	Bilateral	Mucoid	C type	-
15	6	M	Bilateral	Mucoid	C type	-
16	5	M	Bilateral	Mucoid	R=B type L=C type	-
17	7	F	Bilateral	Mucoid	B type	-
18	3	M	Bilateral	Mucoid	B type	-
19	9	M	Bilateral	Mucoid	C type	-
20	7	M	Bilateral	Mucoid	R=B type L=C type	-
21	2	M	Unilateral	Mucoid	B type	-

M: Male, F: Female, R: Right, L: Left

## Discussion

The main cause of the stomach infection in human and primates is Helicobacter pylori (spiral-shaped, Gram negative, microaerophilic bacteria). Stomach is the specific location for H. pylori and there is no other reservoir place for it. The route of the infection has not been defined clearly and fecal-oral, oral-oral, and iatrogenic routes are the three possibilities. Without any treatment, when the microorganism enters the body, its presence continues for lifelong. H. pylori infection is closely related to the chronic active gastritis, gastric and duodenal ulcer, gastric lymphoma, and gastric cancer for human beings. The relationship between H. pylori and gastroesophageal reflux was not defined clearly. But some studies show that, the elimination of H. pylori has no effect on reflux disease or it actually increases it.^5^

In several studies, the relationship between H. pylori and the pathogenesis of various upper aerodigestive tract problems have been shown. In patients with chronic sinusitis, significant increasing of H. pylori in the nasal mucosa was detected by PCR.^6^ There is an association between chronic middle ear problems and gastroesophageal reflux. Higher concentration of pepsin/pepsinogen (×1000) in the middle ear than serum of patients with OME has been reported by Tasker et al.^3^ In this study, we applied PCR for the detection of H. pylori by specimen collection from OME. Bacteriological studies of OME using highly sensitive molecular biology techniques, such as polymerase chain reaction, have demonstrated that the traditional culturing methods are inadequate to detect many viable bacteria present in OME.^7^ Therefore, we performed PCR technique for H. pylori detection technique to collect specimens.

In the present study, among 40 ears from 21 patients that were tested with the PCR, no evidence of H. pylori was detected in the middle ear effusion of the patients with OME. However, Fancy et al.^2^ showed that 32 percent of middle ear aspirates were positive for Helicobacter pylori. In a report by Agirdir et al.,^8^ amongst 30 OME patients, CLO testing was positive in the middle ear effusions for 66.6% (n=20) of the patients. Furthermore, the adenoids of the patient group and the control group showed no significant difference in the prevalence of H. pylori. Yilmaz et al.^9^ reported that the PCR and culture of the effusion in the middle ear tested positive for H. pylori in 45% of the OME patients. Using PCR, Karlidag et al.^10^ showed that among 55 ears, the effusion in 9 ears (16.3%) was positive for H. pylori. It has been proposed that, reflux of gastric contents through the pharynx can lead to transfer of H. pylori into the middle ear, adenoid and tonsillar tissue.^11^ Mucosal inflammation and oedema, due to acid reflux in the nasopharynx, may interfere with eustachian tube normal function; therefore, nasopharyngeal bacteria can enter into the middle ear. For example, Helicobacter pylori is one of entering bacteria. Adenoid tissue may act as a source of infection that in patients with OME, H. pylori could disseminate via the eustachian tube to the middle ear.

However, in our study, we did not perform any test on adenoids of our patients and we could not compare the relationship between adenoid and OME in the present study. Note that, in a study by Bitar et al., they failed to detect H. pylori by either culture or polymerase chain reaction, and concluded that H. pylori plays no part in OME in children, and does not colonise adenoid tissue.^12^ In addition, Sudhoff et al. in a critical review showed a poor evidence for the existence of H. pylori-associated otitis media with effusion.^7^ This study also supports our findings that there was no evidence for the presence of H. Pylori in OME patients. As reported by Morinaka et al.,^13^ while the bulk of the OME samples were positive in terms of H. pylori’s presence, however, merely 3 out of the 13 samples displayed viable organisms. This shows that, although there may be reflux of gastric juice into the nasopharynx or middle ear, this in itself fails to prove that H. pylori can survive in the middle ear and influence its physiology. In addition, the pH value of middle-ear effusions in chronic OME patients was found to be between 7.0 and 9.0. Such an alkaline environment will allow H. pylori to survive; however, its growth will be impeded. Helicobacter pylori requires an acidic environment in order to survive in the presence of urea.^14^ Therefore, in our study, we could not detect H. Pylori by PCR.

A study by Post^15^ showed the presence of pathogens attached to the middle-ear mucosa as a bacterial biofilm rather than as free-floating organisms in a middle-ear effusion. Probably in our study, there was no H. pylori organism in free-floating form and thus could not be detected by PCR.

## Conclusion

In this study, it is possible that there was no H. pylori organism in free-floating form and consequently it could not be detected by PCR. 
